# Microstructure and Mechanical Properties of a Medium-Mn Steel with 1.3 GPa-Strength and 40%-Ductility

**DOI:** 10.3390/ma14092233

**Published:** 2021-04-26

**Authors:** Shaobin Bai, Wentao Xiao, Weiqiang Niu, Dazhao Li, Wei Liang

**Affiliations:** 1 College of Materials Science and Engineering, Taiyuan University of Technology, Taiyuan 030024, China; sh1989bedu@163.com (S.B.); wentao971107@163.com (W.X.); niuweiqiang002021@163.com (W.N.); 2 College of Materials Science and Engineering, North University of China, Taiyuan 030051, China; dazhaoli@163.com

**Keywords:** medium-Mn steel, partition behavior, TRIP effect, precipitation strengthening, aging process

## Abstract

Steel designs with superior mechanical properties have been urgently needed in automotive industries to achieve energy conservation, increase safety, and decrease weight. In this study, the aging process is employed to enhance the yield strength (YS) by tailoring the distribution of V-rich precipitates and to improve ductility by producing high volume fractions of recrystallized ferrite in cold-rolled medium-Mn steel. A reliable method to acquire ultra-high strength (1.0–1.5 GPa), together with ductility (>40%), is proposed via utilizing non-recrystallized austenite and recrystallized ferrite. Similarly to conventional medium-Mn steels, the TRIP effect, along with the mild TWIP effect, is responsible for the main deformation mechanisms during tensile testing. However, the coupled influence of precipitation strengthening, grain refinement strengthening, and dislocation strengthening contributes to an increase in YS. The studied steel, aged at 650 °C for 5 h, demonstrates a YS of 1078 MPa, ultimate tensile strength (UTS) of 1438 MPa, and tensile elongation (TE) of 30%. The studied steel aged at 650 °C for 10 h shows a UTS of 1306 MPa and TE of 42%, resulting in the best product in terms of of UTS and TE, at 55 GPa·%. Such a value surpasses that of the previously reported medium-Mn steels containing equal mass fractions of various microalloying elements.

## 1. Introduction

A reduction in vehicle weight has become increasingly urgent in order to reduce exhaust emissions and improve fuel efficiency. In addition to meeting lightweight requirements, an outstanding combination of strength and ductility for automotive steels is necessary in order to ensure passenger safety [1,2]. Some structural reinforcement mountings, such as bumpers and pillars, require an ultra-high YS (>1.0 GPa). Currently, cold rolling and hot-press forming processes are mainly employed to manufacture Giga-grade automotive sheets. The hot-press forming process alone has been successfully adopted in the production of 1.3 GPa-grade sheets [3]. However, it is limited in application due to the energy consumption induced by heating procedures and poor productivity resulting from prolonged process times [4]. Therefore, cold-rolled 1.3 GPa-grade sheets with preeminent surface quality and formability are urgently needed to address the drawbacks of hot-press forming steels.

Austenite and ferrite + austenite microstructures are considered the optimal candidates for developing a cold-rolled 1.3 GPa-grade sheet due to the occurrence of transformation-induced plasticity (TRIP), twinning-induced plasticity (TWIP), and microband-induced plasticity (MBIP), respectively [5,6,7,8,9,10]. TWIP steels show ideal UTS (>1.0 GPa) and TE (>60%), but offer poor YS (0.2–0.6 GPa) due to austenite matrix characteristics [10]. Therefore, a microstructure with full austenite might not be the best choice. In ferrite + austenite duplex steels, the sustainable transformation from austenite to martensite during tensile testing greatly contributes to their excellent mechanical properties. To further optimize the mechanical properties, grain refinement strengthening [10] and precipitation strengthening [6,7] have been employed to improve the YS. Through these methods, strength is significantly enhanced, but the ductility is greatly limited. Therefore, the question of how to ensure the improvement of strength in medium-Mn steels while achieving the ideal ductility is expected to be addressed in the future. It was found that TRIP and TWIP effects were the main reason for the enhancement of both strength and ductility in medium-Mn steels [7,9]. During tensile testing, austenite with poor mechanical stability preferentially transforms into martensite; whereas austenite with high mechanical stability can generate nano-mechanical twins. In other words, the occurrence of TRIP and TWIP effects is closely related to austenite volume fraction and mechanical stability [2,5]. Austenite with the optimal volume fraction and mechanical stability is the best choice. However, it is not easy to find the equilibrium between the volume fraction and mechanical stability of austenite. It is well known that cold-rolled sheets are easily recrystallized during long-duration annealing, and the non-recrystallized regions seriously limit the ductility [8,9,10]. Thus, a promising method to manufacture steel of ultra-high strength (1.0–1.5 GPa) and outstanding ductility (>40%) is the utilization of non-recrystallized austenite and recrystallized ferrite in a cold-rolled medium-Mn steel (Fe-10Mn-2Al-0.4C-0.6V, by wt.%). Therein, the non-recrystallized austenite with varied mechanical stability can sustainably provide the TRIP effect to strengthen the medium-Mn steels, and the recrystallized ferrite can undergo sustainable plastic deformation to enhance ductility.

In the studied cold-rolled medium-Mn steel, an aging process is employed to enhance the YS by tailoring the distribution of V-rich precipitates (abbreviated as VC) and to improve the ductility by producing high volume fractions of recrystallized ferrite. Our results demonstrate that such a promising method can be reliably used to acquire a medium-Mn steel, which shows a strength of 1.3 GPa and a ductility of 40%. The delicate relationship between the evolution of a microstructure containing VC precipitates and the steel mechanical properties are elaborated in detail.

## 2. Materials and Experiments

**Figure 1:**
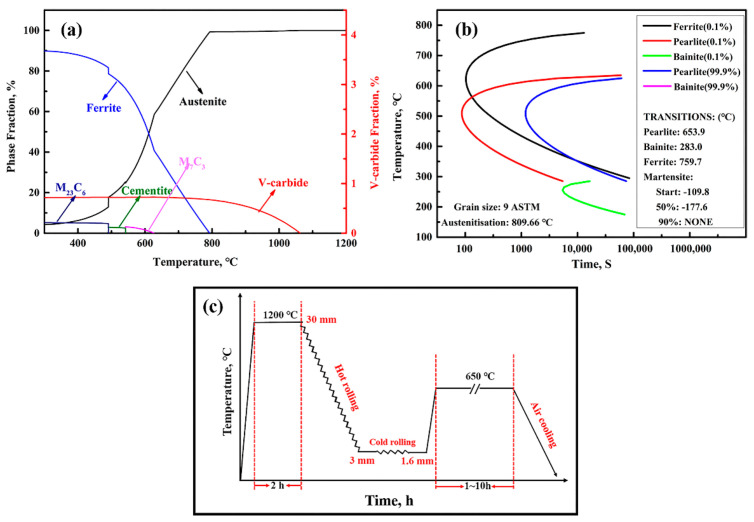
The results calculated using JMatPro software (**a**,**b**) and the abstract process flow diagram (**c**). (**a**) The calculated phase fraction as a function of temperature in the present steel; (**b**) the calculated TTT curves of the studied steel; (**c**) the abstract process flow diagram of the rolling and aging processes of the present steel.

The studied steel, of which the chemical composition is Fe-10Mn-2Al-0.4C-0.6V (wt.%), was melted in a vacuum induction furnace and then cast into a 50-kg square billet. Its equilibrium phase volume fractions at various temperatures were simulated using JMatPro software (Figure 1a). The A_3_ value was 800 °C, and the temperature of the 50% austenite phase fraction was 615 °C. Cementite and VC dissolved at 620 °C and 1060 °C, respectively. The corresponding TTT curves of the studied steel are shown in Figure 1b. These simulated temperatures have an important guiding role in the subsequent heat-treatment process.

The rolling and aging processes are illustrated in Figure 1b. After homogenization at 1200 °C for 2 h to ensure both microstructure uniformity and the complete dissolution of VC particles, a 30-mm-thick strip was hot-rolled to 3 mm thickness over seven passes between 1050 °C and 800 °C before being air-cooled to room temperature. A 3-mm-thick hot-rolled sheet was rolled to make a 1.6 mm thickness sheet over three passes at room temperature. The cold-rolled sheets with 1.6 mm thickness were aged at 650 °C for 1 h, 5 h, and 10 h (referred to as I-1 h, I-5 h, and I-10 h, respectively), and then air-cooled to room temperature. According to the results calculated using JMatPro software 8.0 (Sente Software, Guildford, UK), cementite will be completely dissolved at 650 °C, because it is higher than the required 620 °C (Figure 1a).

The samples for the electron probe micro-analyzer (EPMA, JEOL JXA-8503F, JEOL, Tokyo, Japan) and scanning electron microscope (SEM, JEOL JXA-8503F) were etched in a mixed acid solution (4% HNO_3_ and 96% C_2_H_5_OH with a current of 20 V for 60 s) after mechanical polishing. Energy dispersive spectrometry (EDS) was employed to analyze the partition behavior of Mn and Al between austenite and ferrite phases of all aged samples. Then, the corresponding C content in austenite of a given sample could be calculated using Equation (1) [11,12]:

(1) $$a_{\gamma }=3.556+0.0453X_{C}+0.00095X_{\mathrm{Mn}}+0.0056X_{\mathrm{Al}}$$

where *α*_γ_ represents the lattice parameter of austenite, which was able to be measured from the (220) diffraction peak according to X-ray diffraction (XRD).

The Vickers hardness test was carried out on the mechanically polished sample in an HMAS-D1000SMZ machine (Yanrun Optical Machinery Technology Co., Ltd., Shanghai, China) with a load of 0.1 kgf and a loading time of 15 s. Seven data points were measured for a given sample, and the average values were calculated. The thin foils for transmission electron microscopy (TEM, Tecnai G20, FEI, Hillsboro, OR, USA) studies were mechanically polished down to a thickness of 50 μm using a twin-jet machine with the above-mixed solution at −25 °C. The elemental make-up of the VC precipitates was determined by means of EDS attached to the TEM system. EBSD measurements (step size: 0.15 μm) were executed after electrolytic polishing in a mixed acid solution (10% HNO_3_ and 90% C_2_H_5_OH with a current of 30 V for 40 s) at −20 °C.

The samples for XRD studies were electrochemically polished in the mixed solution to eliminate surface stress; then, the XRD experiment was carried out using Cu-Kα radiation with a speed of 5°/min. The measured integral strength of the diffraction peaks (*γ*(200), *γ*(220), *γ*(311), *α*(200), and *α*(211)) was employed to calculate the austenite fraction according to Equation (2) [13]:

(2) $$V_{\gamma }=1.4I_{\gamma }/\left(1.4I_{\gamma }+I_{\alpha }\right)$$

where *V*_γ_ is the austenite volume fraction, and *I*_γ_ and *I*_ɑ_ are the integrated intensities of the FCC and BCC diffraction peaks, respectively.

Samples of 12.5 mm width and 25 mm length for tensile tests were machined from the annealed sheets along the rolling direction. The tensile tests were performed on a WDW-200 tensile testing machine (Kexin Test Instrument Co., Ltd., Changchun, China) with a speed of 0.5 mm/min to obtain the tensile properties at room temperature, and the results were the average of three samples. The interrupted tensile tests were employed to further analyze the microstructure evolution during tensile testing. The 0.2% offset strength was considered the final YS.

## 3. Results

### 3.1. Microstructure

#### 3.1.1. Microstructure of Cold-Rolled Medium-Mn Steel

**Figure 2:**
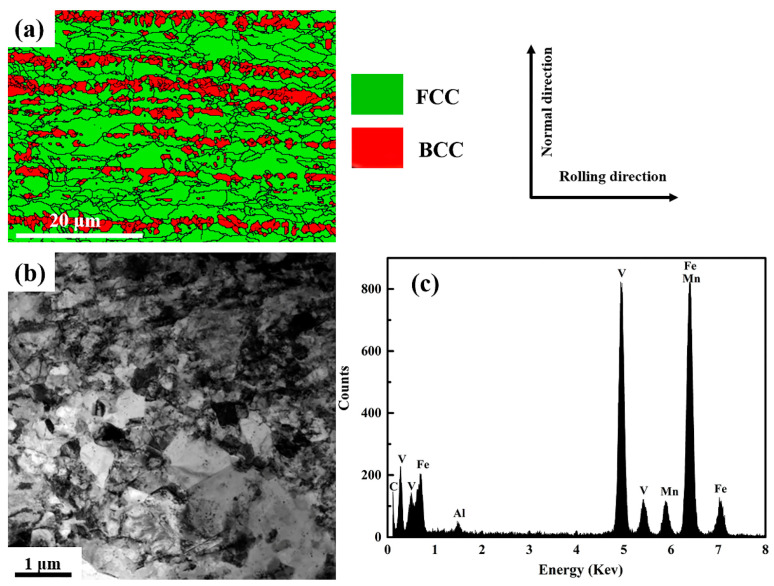
Microstructure characterization of samples after cold rolling. (**a**) EBSD phase image; (**b**) TEM image; (**c**) the STEM-EDS result for the VC precipitates (yellow arrows).

Figure 2 depicts the microstructure of the present medium-Mn steel after cold rolling. The volume fractions of BCC and FCC in the cold-rolled sample are 65% and 35%, respectively (Figure 2a). Some banded ferrite/martensite and equiaxed austenite structures along the rolling direction were observed, as shown in Figure 2a. Moreover, a large amount of ultrafine precipitates (the yellow arrows) was observed in the phase boundaries and grain interior (Figure 2b). Combined with the STEM-EDS results in Figure 2c, the precipitates can be identified as V-rich precipitates.

#### 3.1.2. Microstructure of Aged Medium-Mn Steel

**Figure 3:**
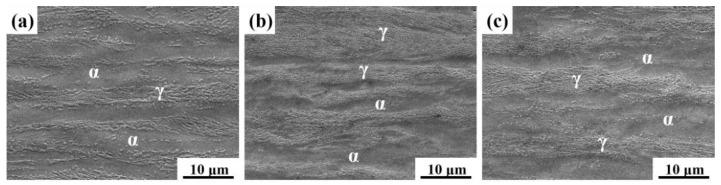
SEM microstructure of samples after the aging process. (**a**) I-1h; (**b**) I-5h; (**c**) I-10h.

Figure 3 shows the SEM microstructure of samples after the aging process. The microstructures of the aged samples were composed of austenite and ferrite phases. With the increasing aging time, the austenite volume fraction first increased to a peak in sample I-5 h, and then decreased in sample I-10 h.

**Figure 4:**
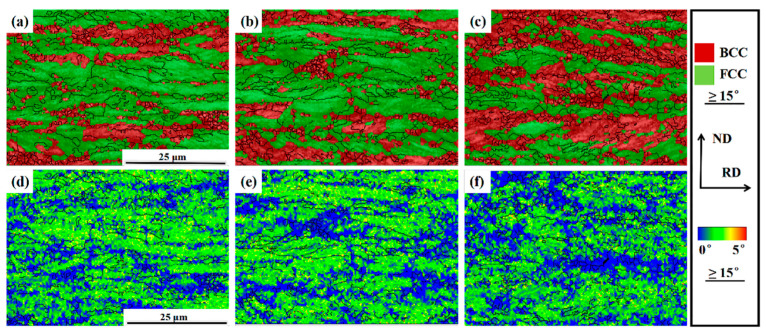
The EBSD phase images (**a**–**c**) and the corresponding KAM results (**d**–**f**) of aged samples, respectively. (**a**,**d**) I-1 h; (**b**,**e**) I-5 h; (**c**,**f**) I-10 h. RD and ND represent rolling direction and normal direction, respectively.

The EBSD phase images of samples subjected to 650 °C aging treatment are displayed in Figure 4a–c. They are examined at the position along the RD-ND (rolling direction-normal directions) plane. A few ultrafine ferrite grains with a size of ten nanometers can be observed in the microstructures, embedded in the coarse-banded austenite and between the austenite interfaces (Figure 4a–c). In addition, a large amount of equiaxed ferrite grains can be observed in all samples, especially in sample I-10 h (Figure 4c). Such a result may be attributed to the recrystallization behavior of ferrite during the aging process [6].

EBSD kernel average misorientation (KAM) maps of samples I-1 h, I-5 h, and I-10 h are shown in Figure 4d–f. KAM maps are considered a measure of strain-induced local orientation gradients [7]. The maximum misorientation angle is 5°. With increasing aging time, the blue regions showed a small increase (<1°) in the KAM values. Austenite grains showed high KAM values (Figure 4d), whereas ferrite grains showed low KAM values (Figure 4e,f). High KAM-value regions containing elongated grains and low KAM-value regions containing equiaxed grains can be defined as non-recrystallized and recrystallized regions, respectively. However, the ferrite grains incompletely recrystallized in sample I-1 h. With increasing annealing temperature, most ferrite grains were recrystallized in samples I-5 h and I-10 h. Especially in sample I-10 h, a high volume fraction of recrystallized ferrite grains led to a low KAM value and a decrease in the mean ferrite grain size.

The average grain sizes of samples I-1 h, I-5 h and I-10 h were 0.298 μm, 0.312 μm, and 0.353 μm, respectively. Some fresh austenite grains, which were transformed from ultra-fine ferrite grains during annealing/aging, were close in size [6]. In contrast, a few coarse austenite grains (>5 μm) were present in the samples. This could be because the original coarse martensite transformed into austenite during aging, or the original coarse austenite grains were retained during cold rolling, and then coarsened during the long-duration aging treatment. Such austenite grains with a bimodal size distribution, which could transform into martensite or twins during tensile testing, play an important role in determining the steel microstructure evolution and mechanical properties [14]. In summary, the previous martensite/ferrite banded structure, whose grains can be refined through either recrystallization or transformation into austenite grains during the aging/annealing treatment, plays an inestimable role in the subsequent microstructure evolution.

**Figure 5:**
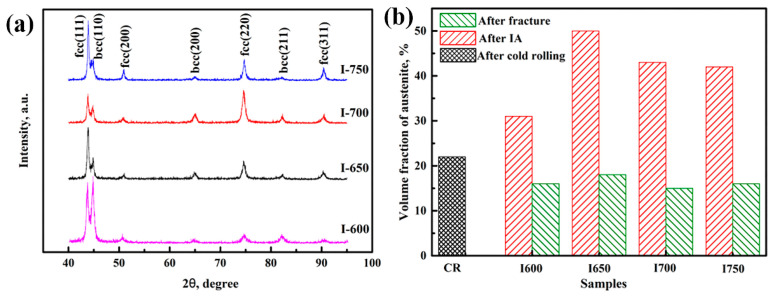
(**a**) The XRD results of samples after aging for various times and (**b**) the calculated austenite volume fraction and austenite transformation ratio of the aged samples before and after tensile testing. CR represents cold rolling.

Figure 5 shows the XRD result of samples after aging and the calculated results of austenite volume fractions and transformation ratios before and after tensile testing. The volume fraction of austenite increased significantly from 59% in sample I-1 h to 71% in sample I-5 h, and then decreased to 52% in sample I-10 h.

#### 3.1.3. Microstructure Characterization of VC Precipitates

**Figure 6:**
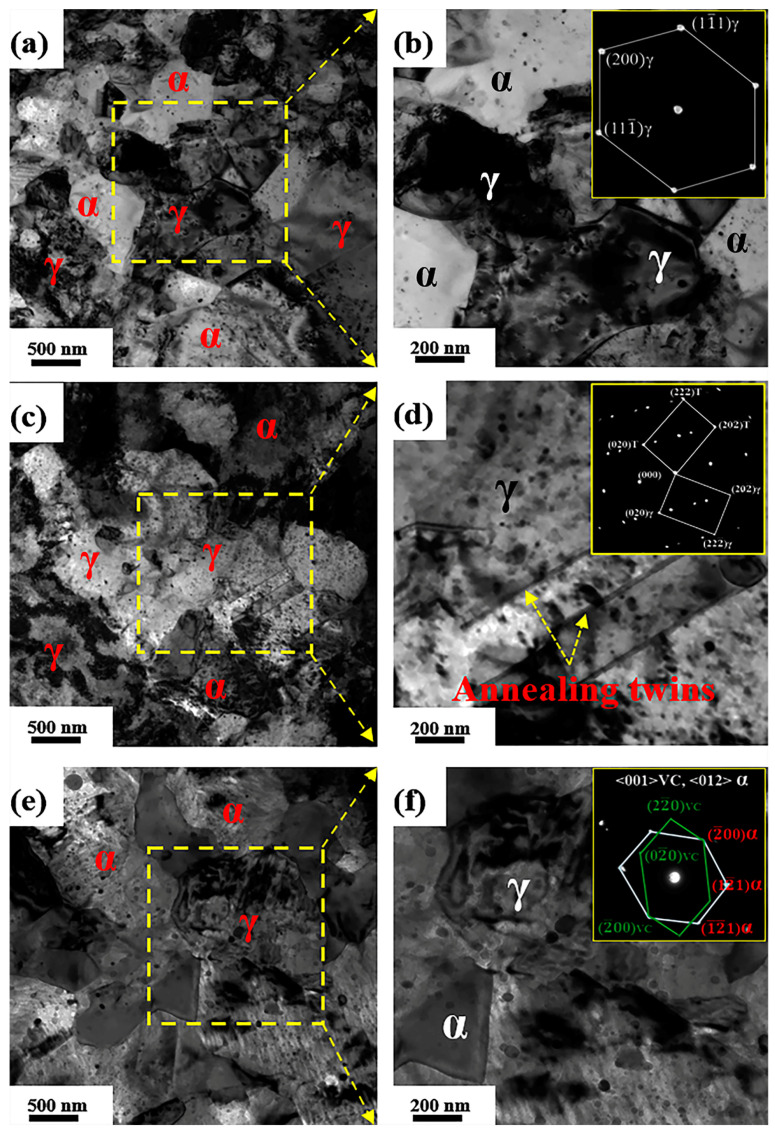
TEM images of the aged samples. (**a**,**c**,**e**) TEM images of austenite and ferrite of sample I-1 h, I-5 h, and I-10 h, respectively; (**b**,**d**,**f**) The enlarged images of marked regions in (**a**,**c**,**e**), respectively. The insets in (**b**,**d**,**f**) are the SAED patterns of austenite, annealing twins, and VC precipitates, respectively.

The distribution of nanoscale VC precipitates in ferrite and austenite and the microstructures of samples after the aging process are shown in Figure 6. VC particles precipitated along the grain boundaries or dislocation interfaces. Some ferrite grains, with sizes of 80–500 nm, were observed either between (Figure 6a,e) or within the austenite grains (Figure 6c). Such ferrite grains are defined as intra-granular ferrite (IGF) grains. Dense dislocation forests were observed in the austenite and ferrite grains (Figure 6a,c), especially near the austenite grain boundaries (Figure 6c), suggesting that a large rolling deformation was initially concentrated at the boundaries during cold rolling. Interestingly, some annealing twins were observed in the austenite of sample I-5 h (Figure 6c,d); similar results have not been reported in medium-Mn (Fe-10Mn) steel [15,16]. Twins, as a well-known strengthening mechanism, can refine austenite grains to enhance work hardening. However, some relatively coarse VC particles (>100 nm) precipitated in austenite rather than in ferrite, indicating that VC particles preferentially grow in austenite grains. Relatively ultrafine V-carbide particles (<30 nm) were observed in IGF grains (Figure 6e,f). Such a result may be attributed to the supersaturation of the solute C and V atoms in ferrite grains.

**Figure 7:**
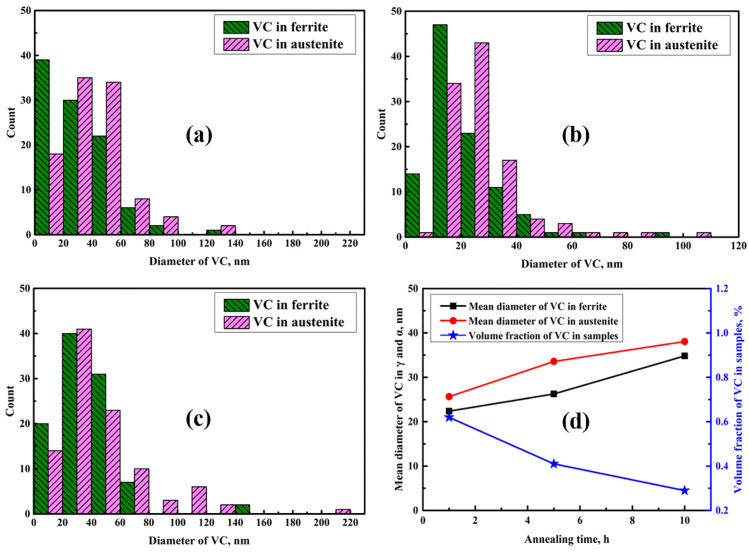
The measured size distributions of VC particles in sample I-1 h (**a**), I-5 h (**b**), and I-10 h (**c**). (**d**) Both the mean diameters and volume fractions of VC particles in samples after aging for various times.

Since VC precipitated in ferrite and austenite simultaneously during annealing, the overall precipitation behavior of VC particles can be reliably estimated. Here, 10 different TEM images were used to carefully analyze the evolution of VC inside the austenite and ferrite phases of samples, as shown in Figure 7. In sample I-1 h, the mean diameters of VC particles in austenite and ferrite ranged from 6.4–109.6 nm and 3.6–88.7 nm, respectively, and the mean diameter of VC particles in this sample was 24.08 nm (Figure 7a). Moreover, the sizes of the VC particles in austenite and ferrite were concentrated at 45 nm and 6 nm (Figure 7a), respectively. As the aging time increased to 5 h, the mean diameter of VC particles increased to 29.07 nm (Figure 7b). Upon further increasing the aging time to 10 h, the mean diameter of VC particles substantially increased to 36.36 nm, as the diameter of the VC particles increased, concentrated in the range of 20–50 nm (Figure 7c). However, the maximum size of VC in austenite was approximately 216 nm (Figure 7c). Moreover, both the volume fraction and mean diameter of VC particles in ferrite and austenite were measured while ignoring the inhomogeneous distribution, as shown in Figure 7d. Obviously, the mean diameters of VC particles in both austenite and ferrite increased with increasing aging time. The coarsening speed of VC in austenite was much faster than that in ferrite (Figure 7d). In addition, the volume fractions of VC particles in the samples continuously decreased with increasing aging time (Figure 7d).

**Figure 8:**
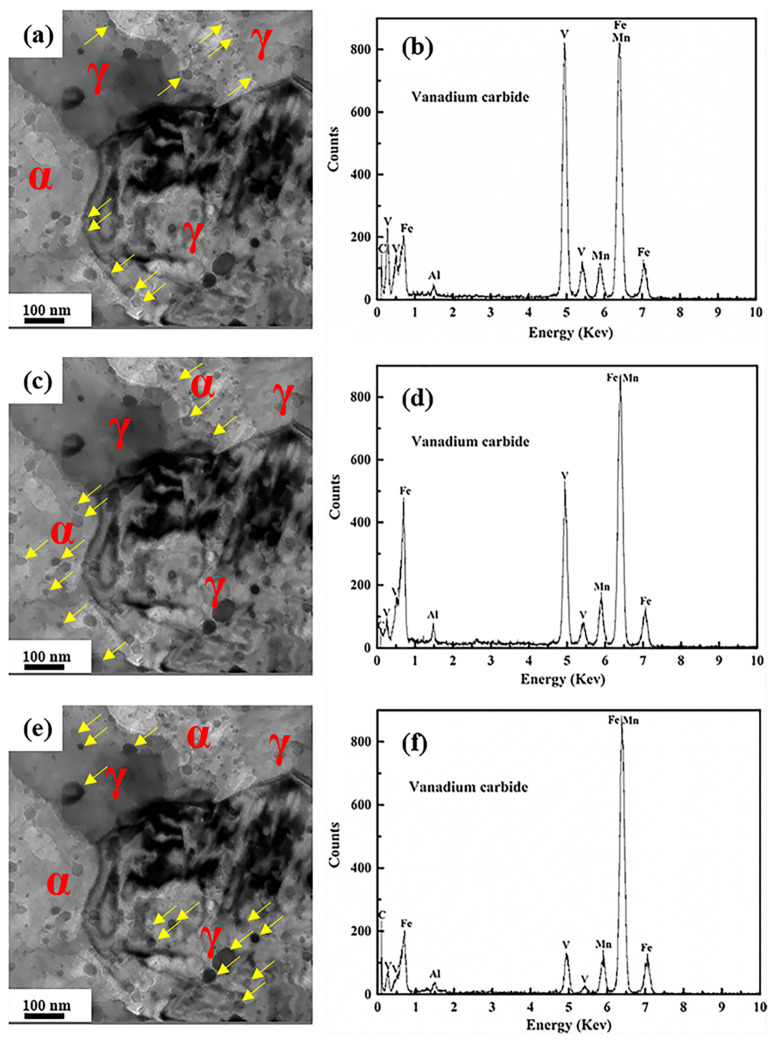
STEM-EDS results of VC precipitates in sample I-10 h. (**a**,**b**) The VC precipitates in phase boundaries; (**c**,**d**) the VC precipitates in ferrite; (**e**,**f**) the VC precipitates in austenite.

The TEM-EDS results of sample I-10 h are shown in Figure 8. The VC particles formed at phase boundaries had the highest V content (Figure 8b). This result may be why the ferrite–austenite boundaries are the necessary path for element diffusion during the aging process, resulting in the greater accumulation of V and C at the boundaries. In addition, grain boundaries are considered the most suitable nucleation sites for precipitates. Therefore, the highest V content was generated at the ferrite-austenite boundaries. However, C (as the austenite stabilizer) was preferentially assigned to austenite during annealing, and more abundant C was available in that phase for the formation of VC precipitates. In addition, grain refinement can absolutely overcome the reduced C concentration in stabilizing austenite [16]. Therefore, V addition can not only refine the grain size, but also enhance the austenite stability; a similar result has been reported in [17].

### 3.2. The Partition Behaviour of Mn and Al between Austenite and Ferrite

**Figure 9:**
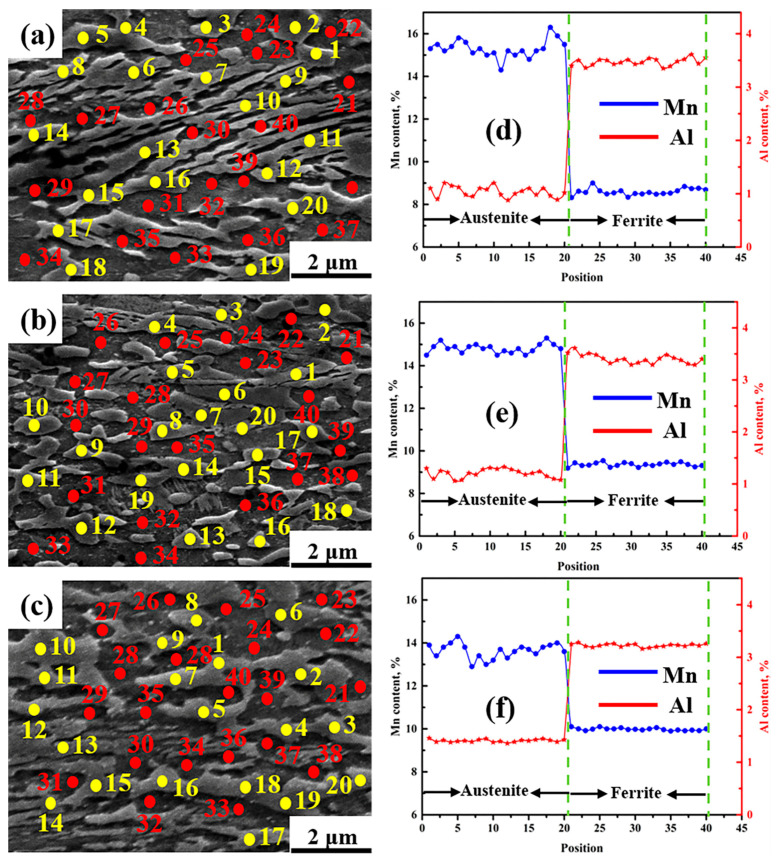
The SE images (**a**–**c**) and the measured partition behavior of Mn and Al in samples (**d**–**f**). (**a**,**d**) I-1 h; (**b**,**e**) I-5 h; (**c**,**f**) I-10 h.

VC particles are generated during the aging process [6,17], wheereas the martensite-to-austenite reverse transformation also occurs [18], accompanied by prominent C, Mn, and Al partitioning behavior [19]. The austenite stability is mainly determined by the chemical composition [20]. Al is considered a stabilizer for ferrite. C and Mn can effectively enhance austenite stability. In this study, EPMA-EDS analysis (using 40 randomly selected points for ferrite and austenite in each sample) was applied to all samples to further analyze the partition behaviors of Mn and Al between austenite and ferrite, as shown in Figure 9. Austenite, which is marked with yellow circles, is numbered from 1–20; whereas ferrite is marked with red circles numbered from 21–40. With increasing aging time, the Mn content in austenite persistently decreased, whereas the Al content in austenite weakly increased, implying that Mn and Al partitioning behavior occurred during the aging process. The longer the aging time was, the lower the Mn content in austenite, resulting in poor austenite stability. It is well known that the C content in austenite cannot be measured accurately using EPMA-EDS. Thus, it should be calculated using Equation (1) combined with the XRD data. The measured C content of samples I-1 h, I-5 h, and I-10 h were 0.75, 0.63, and 0.54, respectively, suggesting that the C content gradually decreased with increasing aging time. Similar results have been reported in V-free medium-Mn steels [17].

### 3.3. Tensile Properties and Work-Hardening Behaviour

**Figure 10:**
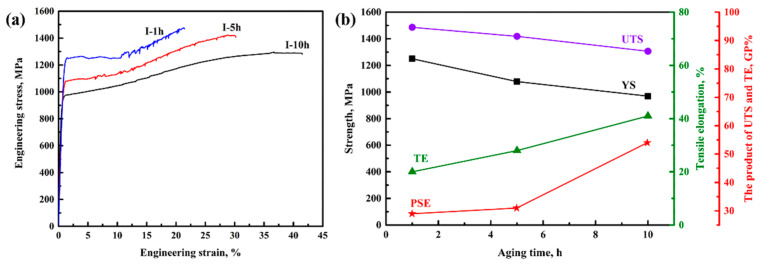
Tensile properties of aged samples. (**a**) Engineering stress–strain curves of aged samples after tensile testing at room temperature; (**b**) the dependence of UTS, YS, TE, and the PSE (the product of UTS and TE) of samples after aging at 650 °C for various times.

Figure 10a shows the engineering stress–strain curves of samples after tensile testing, and the corresponding tensile properties are displayed in Figure 10b. Unlike other samples, sample I-10 h featured no significant serration in the stress–strain curve. The YS, UTS, and TE of sample I-1 h were 1258 MPa, 1486 MPa, and 20%, respectively. When the aging time was increased to 10 h, the YS and UTS gradually decreased to 969 MPa and 1306 MPa, respectively, whereas the TE significantly increased to 42%. Interestingly, sample I-5 h presented a greatly improved TE value, increasing from 20% to 30% with little loss of UTS (–50 MPa), compared to sample I-1 h. Moreover, the products of UTS and TE for samples I-1 h, I-5 h, and I-10 h were 30 GPa·%, 44 GPa·%, and 55 GPa·%, respectively, which satisfy the automotive industry requirements. Therefore, the aging process can be employed to optimize the tensile properties of medium-Mn steels, with good strength retainment and plasticity enhancement. Such an outstanding combination of mechanical properties may be closely associated with the evolution of both VC particles and austenite during the aging process.

**Figure 11:**
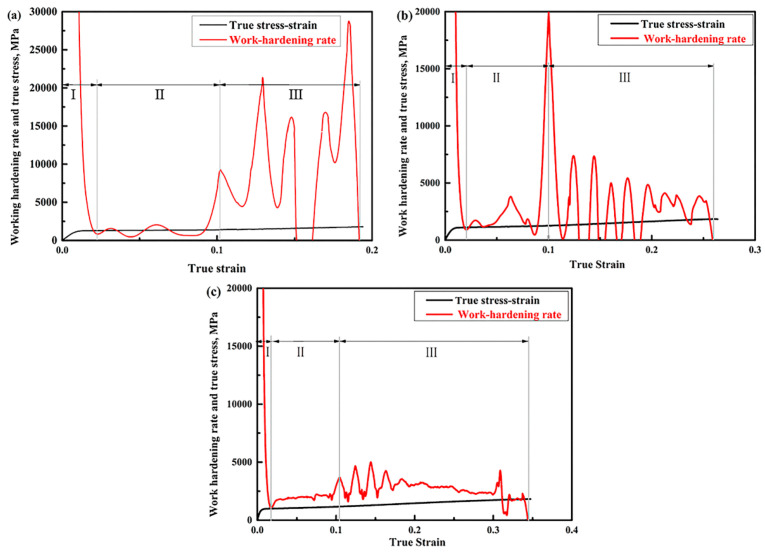
The work-hardening rate–true strain curves (red lines) and true strain–stress curves (black lines) of (**a**) sample I-1 h, (**b**) sample I-5 h, and (**c**) sample I-10 h.

Figure 11 shows the work hardening rate–true strain and true stress–strain curves of the aged samples after tensile testing. A sharp decrease tendency for the work hardening rate was observed in all the samples at the initial strain regime (Stage-I). Then, there were two different serrated fluctuations in the work hardening rate curves (marked with Stage-II and Stage-III). The formation of Stage-Ⅱ is attributed to yield point elongation. Moreover, both the Cottrell atmosphere and the partial TRIP effect result in a low work hardening rate [20]. However, the higher work hardening rate in Stage-III, which presents an extension of an uncertain value, should be attributed to the sufficient transformation of austenite to martensite [21]. Additionally, it is worth noting that the values of fluctuating peaks in the work hardening rate curves of different aged samples are rather inconsistent. Such a result should be attributed to the great differences in samples, such as in the austenite stability and austenite fraction [6,14]. Additionally, both the austenite fraction and austenite stability are the main factors determining the strain-hardening behavior. In other words, the strain-hardening behavior is barely affected by V addition, as compared to a previous report [6].

### 3.4. Fractography

**Figure 12:**
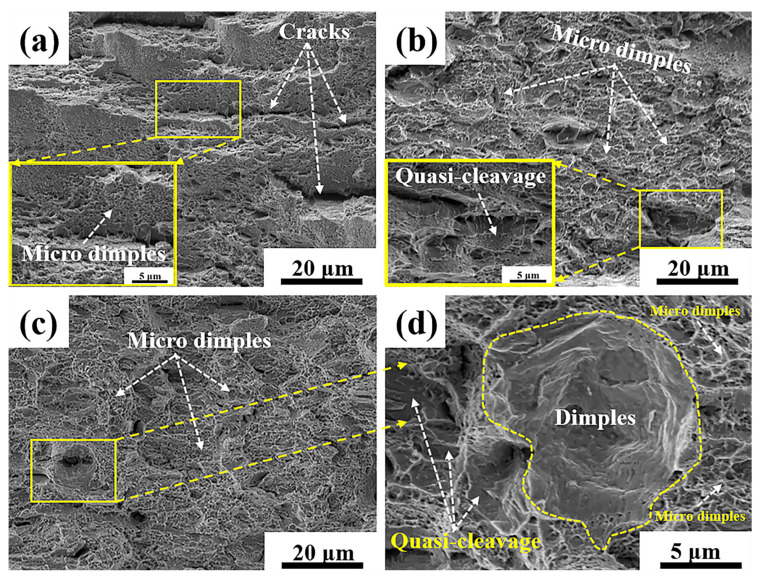
Fracture surface appearance of samples after tensile testing. (**a**) Sample I-1 h; (**b**) Sample I-5 h; (**c**) Sample I-10 h; (**d**) the enlarged images of marked regions in (**c**). The insets in (**a**,**b**) are the enlarged areas of the marked areas.

For the aging time range of 1 h to 10 h, the ductility was gradually enhanced. Figure 12 shows the fracture surface appearance of samples after tensile testing. In contrast, the fracture surface of sample I-1 h was found to possess the most micro-cracks and partial micro-dimples, as shown in Figure 12a. The existence of these cracks may induce poor ductility. More micro-dimples than quasi-cleavage facets were observed in sample I-5 h (the enlarged image of the marked area in Figure 12b). These quasi-cleavage facets imply the occurrence of cleavage-type fractures, leading to poorer ductility. The close examination of the fracture surface revealed that sample I-10 h possessed areas with more extensive micro-dimples than other samples (Figure 12c,d). The presence of a uniform distribution of micro dimples results in the best ductility.

### 3.5. Hardness Measurements

**Figure 13:**
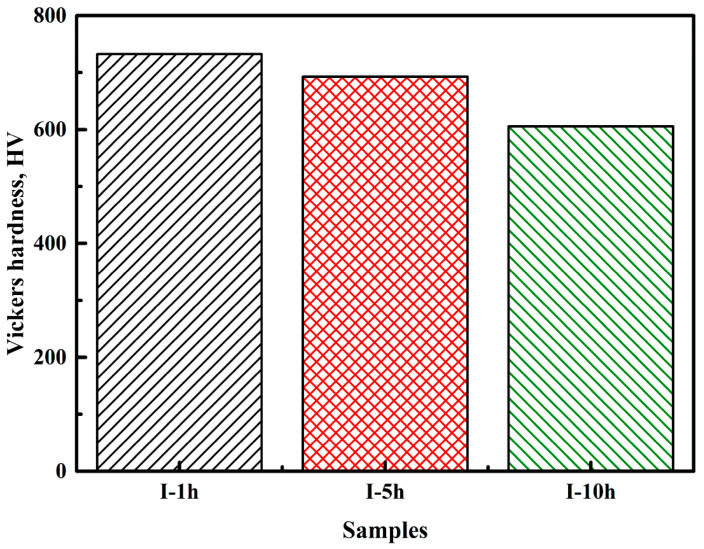
Vickers hardness of samples after aging for various times.

Figure 13 shows the Vickers hardness of aged samples. The Vickers hardness of aged samples gradually decreased with the increasing aging time, and the corresponding values were 732.5 HV0.1, 692.5 HV0.1, and 605.6 HV0.1, respectively. This result was similar to the variational trend in the work hardening rates of the aged samples (Figure 11). Long-duration aging results in a large volume fraction of recrystallized ferrite grains, as shown in Figure 4a–c, thus leading to a decrease in dislocation density in aged samples (Figure 4d–f).

## 4. Discussion

### 4.1. Microstructure Evolution during Aging

Different aging times result in changes in both the morphology and size of ferrite and austenite, especially the austenite volume fraction and austenite stability. The longer the aging time is, the lower the concentration C and Mn are in austenite after the aging process. Long-duration aging will result in the enlargement of austenite grains (Figure 4), some of which will then undergo martensitic transformation during the cooling process due to the poor stability of austenite [22]. Therefore, the volume fraction of austenite first reaches a peak value, and then decreases with a further increase in aging time (Figure 5).

The aging process strongly influences the evolution of VC precipitates due to the partitioning behavior between austenite and ferrite. An uneven distribution of VC precipitates was observed in the samples (Figure 6). Such a result may be attributed to the fact that V and C are not completely dissolved in the matrix [6]. Additionally, the small difference in the crystal lattice between VC and ferrite (Figure 6f), which has been previously reported in [17], may result in a decrease in the nucleation barrier. This decrease may contribute to a higher density of ultrafine VC particles generated in ferrite than in austenite (Figure 7). Note that the strong partitioning behavior of C and V between austenite–ferrite boundaries can immensely accelerate the nucleation of VC particles, due to austenite reversion during the aging process. With a further increase in aging time, the VC particles gradually became coarser via Ostwald ripening due to the high diffusion rate (Figure 7d).

Moreover, C and V are much more easily dissolved in austenite than that in ferrite, although some VC particles can remain in austenite in the studied medium-Mn steel. This result can be attributed to the aging time and subsequent air cooling. A previous study reported that an increase in the heating rate resulted in a decrease in the ordering time available for the formation of precipitate structures, leading to a decrease in the volume fraction of precipitate particles [23]. Similarly, the quick cooling rate can greatly limit the nucleation of VC particles.

It is worth noting that the chemical compositions of VC particles are determined by their nucleated sites. During the aging process, the nucleated sites can be attributed to the simultaneous occurrence of martensite-to-austenite reverse transformation, the partitioning behavior of elements (C, Mn and Al), and VC ordering. On the one hand, grain boundaries can be considered nucleation sites for precipitates [24], whereas on the other hand, phase boundaries between austenite and ferrite are efficient and necessary channels for element diffusion during annealing [1]. This results in a high V content at the phase boundaries. Therefore, the highest V content of VC was presented at the phase boundaries.

### 4.2. Yield Strength

VC can contribute to the improvement of YS through precipitation strengthening [25]. However, during the initial tensile testing, the strength of medium-Mn steel was mainly determined by ferrite deformation. Thus, the relationship between the microstructure evolution in ferrite and the strength of the steel is analyzed here in detail. The YS increments (*σ*_YS_) of the samples are estimated using Equation (3) [6,17,26]:

(3) $$\sigma_{\mathrm{YS}}=\sigma_{D}+\sigma_{G}+\sigma_{P}$$

where *σ*_D_, *σ*_G_, and *σ*_P_ are the YS increments of dislocation strengthening, grain refinement strengthening, and VC precipitation strengthening, respectively. The value of *σ*_D_ can be calculated using Equation (4) [25,27]:

(4) $$\sigma_{D}=M\alpha Gb\sqrt{\rho_{\alpha }}$$

where *M*, *α*, *G*, *b*, and *ρ_α_* are the Taylor factor (2.75), a constant (0.38), the shear modulus (81.6 GPa), the Burgers vector (0.248 nm), and the average dislocation density in ferrite, respectively. According to the Hall–Petch formula, *σ*_G_ can be estimated using Equation (5) [28]:

(5) $$\sigma_{G}=K_{i}/\sqrt{D_{i}}$$

where *K*_i_ and *D*_i_ represent the Hall–Petch factor (17.4 MPa/mm^−0.5^) and average grain size, respectively. When the mean diameter of the VC particles exceeds the critical radius, the medium-Mn TRIP steel is strengthened by precipitation strengthening through Orowan looping [25]. According to the well-known Ashby–Orowan Equation (6) [29], *σ*_P_ is predicted as follows:

(6) $$\sigma_{P}=0.84(\frac{2T}{Rb\sqrt{\frac{2\pi }{3f}}})$$

where *T* = Gb^2^/2 is the line tension and G = 81.6 GPa is the shear modulus for ferrite. *R* and *f* represent the mean diameter and volume fraction of VC particles, respectively. b = 0.248 nm is the Burgers vector.

**Figure 14:**
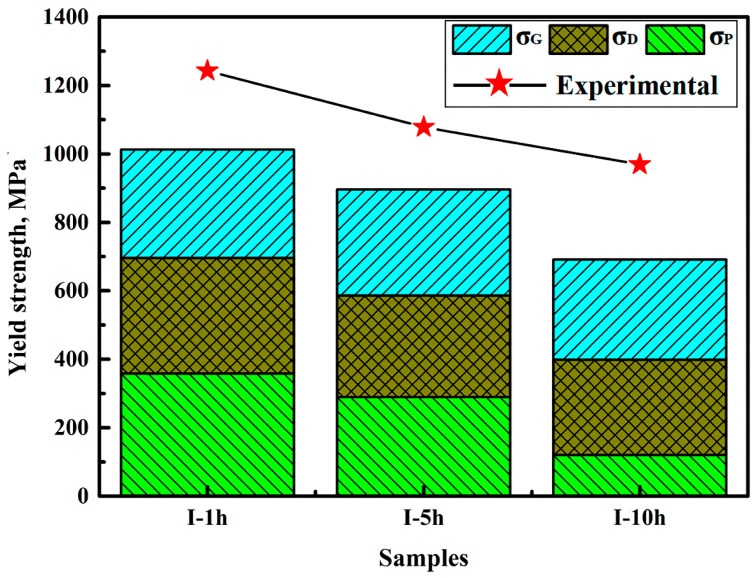
The contributed yield strength increments of dislocation strengthening (*σ*_D_), precipitation strengthening (*σ*_P_), and grain refinement strengthening (*σ*_G_), respectively.

The YS increments of each strengthening mechanism are shown in Figure 14. Obviously, grain refinement strengthening and precipitation strengthening greatly contribute to YS. However, there is still an appreciable difference between the calculated results of YS and the specific values of YS shown in Figure 10. Given the contributions of multiple strengthening mechanisms to the YS increments, solid solution strengthening is likely responsible for the remaining YS increments.

### 4.3. Microstructure Evolution of Sample I-10 h during Tensile Testing

The combination of various deformation mechanisms, such as the TRIP effect, TWIP effect, and dislocation strengthening, results in an excellent combination of strength and ductility in medium-Mn steels [6,14,15]. The presence of VC and annealing twins (Figure 6c) inevitably affects the deformation mechanisms during tensile testing.

**Figure 15:**
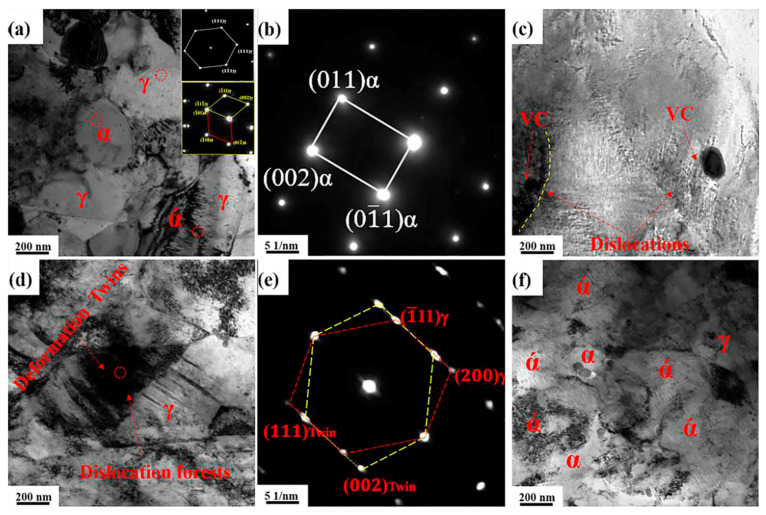
TEM images of sample I-10 h subjected to interrupted tensile testing. (**a**–**c**) Strain to 5%; (**d**–**f**) after fracture. The SAED patterns of the location marked in red circles are shown in (**a**,**b**,**e**).

The TEM microstructure of sample I-10 h subjected to interrupted tensile testing is shown in Figure 15. Both ferrite grains with sizes of 100–250 nm (Figure 15a) and austenite grains with size of 200–300 nm (Figure 15a) are retained after 5% tensile strain. Figure 15a,b depicts the corresponding SAED patterns of ferrite and austenite (marked regions), respectively. Additionally, coarse martensite (>500 nm) can be observed in this sample after 5% tensile strain (Figure 15a), as well as in the fractured sample (Figure 15d,f)). This is because the austenite with poor stability quickly transforms into martensite through the TRIP effect during tensile testing. The SAED patterns at the phase boundaries show typical [111]_ά_ and [110]_γ_ simultaneously, as shown in Figure 15a, implying that the direction of [111]_ά_ is parallel to that of [110]_γ_. Moreover, the diffraction spots of (-11-1)_γ_ correspond to (-101)_ά_, indicating that the direction of (-11-1)_γ_ is parallel to that of (-101)ά. The above results show a typical orientation relationship between austenite and martensite of ((-11-1)_γ_//(-101)_ά_) and [110]_γ_//[111]_ά_). Some VC particles act as a hindrance to dislocation migration, effectively restraining the movement of dislocations and enhancing work hardening [24], as shown in Figure 15c. Nanodeformation twins, together with high-density dislocation forests, can be observed in ultrafine austenite grains (<200 nm), as shown in Figure 15d. Such a result indicates that the ultrafine austenite grains transform into nano-twins through the TWIP effect under large deformation strain, due to their high stability [6]. The corresponding SAED pattern of the deformation twins in austenite is shown in Figure 15e. In Figure 15f, high volume fractions of martensite and partial VC particles can be observed. It is commonly reported that hard phases, such as martensite and precipitates, easily lead to stress concentrations that can induce microcracks [30].

It is well known that ultrafine recrystallized ferrite grains can significantly retard the development of microcracks and effectively delay the failure of specimens. Sample I-10 h had the highest volume fraction of recrystallized ferrite (Figure 4), which could effectively contribute to the improvement of ductility. The occurrence of recrystallization inevitably results in the refinement of grains. Therefore, many refined grains can undergo homogeneous deformation without experiencing stress concentrations, because the deformation energy can be dispersed into fine grains during tensile testing [4]. Ferrite (the soft phase) can undergo sustainable plastic deformation to enhance ductility; however, it cannot provide significant work hardening due to its basic characteristics as the soft phase. Therefore, sample I-10 h showed a larger ductility of 42% and a lower tensile strength of 1306 MPa (Figure 10), resulting in the outstanding product of UTS and TE (55 GPa·%). As the aging time increased, the coarsening of VC precipitates (Figure 7) and the decrease in dislocation density (Figure 14) inevitably resulted in a decrease in YS.

In conclusion, the addition of V can further enhance the mechanical properties of medium-Mn steels due to VC precipitation strengthening, together with a mild TWIP effect, but the low volume fraction of VC particles and the limited number of nano-twins cannot provide sufficient deformation resistance to medium-Mn steel. Consequently, the main strengthening mechanism during tensile testing is still the abundant TRIP effect. Such a result is similar to the deformed behavior in the reported medium-Mn steels without the V element [6,17]. It is obvious that sample I-5 h had the largest austenite transformation ratio of approximately 50% (Figure 5), which suggests a much more obvious TRIP effect over the entire deformation regime.

**Figure 16:**
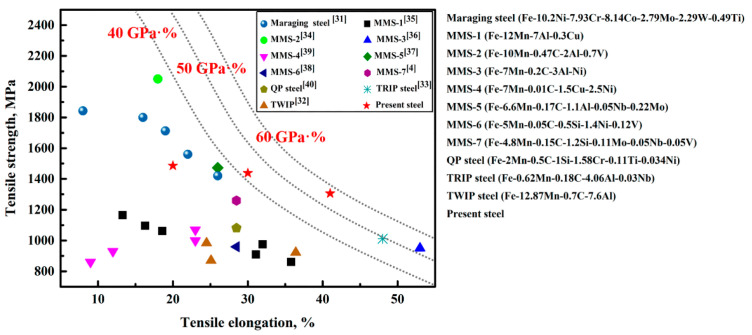
Comparison between the tensile elongation and ultimate tensile strength of the present steel and other steels with various micro-alloying elements reported in References.

A comparison of the tensile strength and TE of other steels with microalloying elements (such as maraging steel [31], TWIP steel [32], TRIP steel [33], medium-Mn steel (MMS) [4,34,35,36,37,38,39], and QP steel [40]) and the present steel is shown in Figure 16. It is obvious that the best combination of the strength and ductility of the studied steel is over 50 GPa·%. Such a value surpasses that of previously reported medium-Mn steel containing the same mass fraction of various microalloying elements.

## 5. Conclusions

In this study, the tensile properties of the present steel were found to be sufficient to meet the requirements of automotive steels. The microstructure evolution and mechanical properties of a medium-Mn TRIP steel containing V aged for various times were analyzed.

1. With increasing aging time, VC particles more easily coarsen in austenite; meanwhile, the volume fraction of VC precipitates in aged samples gradually decreases. V is enriched in austenite–ferrite phase boundaries because phase boundaries are not only nucleation sites for VC precipitates but are also essential for element diffusion during the aging process.
2. The C, Mn, and Al contents in austenite gradually decrease with increasing aging time. Such a varied trend is similar to that of the reported V-free medium-Mn steels.
3. TRIP effect is the main deformation mechanisms in the studied medium-Mn steel, together with a mild TWIP effect during tensile testing. Sample I-5 h demonstrated a YS of 1078 MPa, UTS of 1438 MPa, and TE of 30%. The high YS is mainly attributed to precipitation strengthening and grain refinement strengthening.
4. Sample I-10 h, with the highest volume fraction of recrystallized ferrite (soft phase), showed the largest ductility (42%) and the best PSE value (55 GPa·%). Such a value surpasses that of previously reported medium-Mn steel containing the same mass fraction of various microalloying elements.

## Data Availability

The data presented in this study are availiable on request from the corresponding author. The data are not publicly available due to the data slao forms part of an ongoing study.

## References

[1] Chang-Hyo S., Ki H.K., Kayoung C., Kyung-Hun K. (2012). Deformation behaviour of ferrite-austenite duplex lightweight Fe-Mn-Al-C steel. Scr. Mater..

[2] Sohn S.S., Lee B.J., Lee S., Kim N.J., Kwak J.H. (2013). Effect of annealing temperature on microstructural modification and tensile properties in 0.35 C-3.5 Mn-5.8 Al lightweight steel. Acta Mater..

[3] Jiao Z.B., Luan J.H., Miller M.K., Liu C.T. (2015). Precipitation mechanism and mechanical properties of an ultra-high strength steel hardened by nanoscale NiAl and Cu particles. Acta Mater..

[4] Hao L.H., Ji X., Zhang G.Q., Zhao W. (2020). Carbide precipitation behaviour and Mechanical properties of micro-alloyed medium-Mn steel. J. Mater. Sci. Technol..

[5] Xu W., Rivera-Díaz-del-Castillo P.E.J., Yan W., Yang K., Martín K.D.S., Kestens L.A.I., Zwaag S.V.D. (2010). A new ultrahigh-strength stainless steel strengthened by various coexisting nanoprecipitates. Acta Mater..

[6] Hu B., He B.B., Cheng G.J., Yen H.W. (2019). Super-High-Strength and Formable Medium-Mn steel Manufactured by Warm Rolling Process. Acta Mater..

[7] Sohn S.S., Song H.J., Jo M.C. (2017). Novel 1.5 GPa-strength with 50%-ductility by transformation-induced plasticity of non-recrystallized austenite in duplex steels. Science.

[8] Fonstein N. (2015). Advanced High Strength Sheet Steels.

[9] Somani M.C., Juntunenet P., Karjalainenal L.P. (2009). Enhanced mechanical properties through reversion in metastable austenitic stainless steels. Metall. Mater. Trans. A.

[10] Dini G., Najafizadeh A., Ueji R., Monir-Vaghefi S.M. (2010). Improved tensile properties of partially recrystallized submicron grained TWIP steel. Mater. Lett..

[11] Dijk H.N.V., Zhao A.M.L., Sietsma J., Offerman S.E., Wright J.P. (2005). Thermal stability of retained austenite in TRIP steels studied by synchrotron X-ray diffraction during cooling. Acta Mater..

[12] Blonde R., Jimenez-Melero E., Zhao L., Wright J.P., Dijk N.H.V. (2012). High-energy X-ray diffraction study on the temperature-dependent mechanical stability of retained austenite in low-alloyed TRIP steels. Acta Mater..

[13] Yang F., Luo H.W., Hu C.D., Pu E.X., Dong H. (2017). Effects of intercritical annealing process on microstructures and tensile properties of cold-rolled 7Mn steel. Mater. Sci. Eng. A.

[14] Li J.J., Song R., Li X., Zhou N., Song R. (2019). Microstructural evolution and tensile properties of 70 GPa·% grade strong and ductile hot-rolled 6Mn steel treated by intercritical annealing. Mater. Sci. Eng. A.

[15] He B.B., Luo H.W., Huang M.X. (2016). Experimental investigation on a novel medium-Mn steel combining transformation-induced plasticity and twinning-induced plasticity effects. Int. J. Plast..

[16] He B.B., Huang M.X. (2018). Simultaneous increase of both strength and Ductility of Medium Mn Transformation Plasticity Steel by Vanadium Alloying. Metall. Mater. Trans. A.

[17] Park T.M., Jeong M.S., Jung C. (2021). Improved strength of a medium-Mn steel by V addition without sacrificing ductility. Mater. Sci. Eng. A.

[18] Cai Z.H., Ding H., Misra R.D.K., Ying Z.Y. (2015). Austenite stability and deformation behaviour in a cold-rolled transformation-induced plasticity steel with medium manganese content. Acta Mater..

[19] Chandan A., Bansal G.K., Kundu J., Chakraborty J., Chowdhury S.G. (2019). Effect of prior austenite grain size on the evolution of microstructure and mechanical properties of an intercritically annealed medium manganese steel. Mater. Sci. Eng. A.

[20] Sun B., Fazeli F., Scott C., Guo B., Aranas C., Chu X., Jahazi M. (2018). Microstructural characteristics and tensile behaviour of medium manganese steels with different manganese additions. Mater. Sci. Eng. A.

[21] Cai Z.H., Ding H., Xue X., Jiang J., Xin Q.B., Misra R.D.K. (2013). Significance of control of austenite stability and three-stage work-hardening behaviour of an ultrahigh strength–high ductility combination transformation-induced plasticity steel. Scr. Mater..

[22] Lee S., Lee S.J., Cooman B.C.D. (2011). Austenite stability of ultrafine-grained transformation-induced plasticity steel with Mn partitioning. Scr. Mater..

[23] Rahnama A., Spooner S., Sridhar S. (2017). Control of intermetallic nano-particles through annealing in duplex low-density steel. Mater. Lett..

[24] Zhao J., Zhang F., Chen C., Wang M., Lv B. (2020). Cyclic deformation behaviour of steels with a nanolamellar microstructure and tensile strength of 1500 MPa. Mater. Sci. Eng. A.

[25] Kamikawa N., Sato K., Miyamoto G., Murayama M., Furuhara T. (2015). Stress-strain behaviour of ferrite and bainite with nano-precipitation in low carbon steels. Acta Mater..

[26] Sarkar A., Sanyal S., Bandyopadhyay K.T., Mandal S. (2017). Enhanced strength-ductility relationship in a medium Mn high Al-alloyed multicomponent steel through thermomechanical processing. Mater. Sci. Eng. A.

[27] Bailey J.E., Hirsch P.B. (1960). The dislocation distribution, flow stress, and stored energy in cold-worked polycrystalline silver. Philos. Mag..

[28] Prokoshkin D.A., Vasileva E.V., Lazarev E.M. (1968). Research into the Oxidation of Niobium Alloyed with Vanadium. Titanium, and Zirconium.

[29] Li Y., Li W., Liu W., Wang X., Hua X., Liu H., Jin X. (2017). The austenite reversion and co-precipitation behaviour of an ultra-low carbon medium manganese quenching-partitioning-tempering steel. Acta Mater..

[30] Marinelli M.C., Armas I.A. (2017). Cyclic deformation mechanisms and microcracks behaviour in high-strength bainitic steel. Mater. Sci. Eng. A.

[31] Hu B., Luo H., Yang F., Dong H. (2017). Recent progress in medium-Mn steels made with new designing strategies, a review. J. Mater. Sci. Technol..

[32] Han D., Ding H., Liu D., Rolfe B., Beladi H. (2020). Influence of C content and annealing temperature on the microstructures and tensile properties of Fe-13Mn-8Al-(0.7, 1.2) C steels. Mater. Sci. Eng. A.

[33] Bedekar V., Voothalura R., Yu D.J., Wong A., Nava E.G. (2020). Effect of nickel on the kinematic stability of retained austenite in carburized bearing steels-In-situ neutron diffraction and crystal plasticity modelling of uniaxial tension tests in AISI 8620, 4320 and 3310 steels. Int. J. Plast..

[34] He B.B., Hu B., Yen H.W., Cheng G.J., Wang Z.K., Luo H.W., Huang M.X. (2017). High dislocation density-induced large ductility in deformed and partitioned steels. Science.

[35] Song H.J., Yoo J.S., Kim S.H., Sohn S.S., Lee S.H. (2017). Novel ultra-high-strength Cu-containing medium-Mn duplex lightweight steels. Acta Mater..

[36] Zhang B.G., Zhang X.M., Liu H.T. (2020). Microstructural evolution and mechanical properties of Ni-containing light-weight medium-Mn TRIP steel processed by intercritical annealing. Mater. Sci. Eng. A.

[37] Suh D.W., Kim S.J. (2017). Medium Mn transformation-induced plasticity steels: Recent progress and challenges. Scr. Mater..

[38] Gao G.H., Liu R., Wang K., Gui X.L., Misra R.D.K., Bai B.Z. (2020). Role of retained austenite with different morphologies on sub-surface fatigue crack initiation in advanced bainitic steels. Scr. Mater..

[39] Li Y., Li W., Xu C., Min N., Liu W., Liu H. (2020). Investigation of hierarchical precipitation on bimodal-grained austenite and mechanical properties in quenching-partitioning-tempering steel. Mater. Sci. Eng. A.

[40] Lu J., Yu H., Kang P., Duan X.N. (2018). Study of microstructure, mechanical properties and impact-abrasive wear behaviour of medium-carbon steel treated by quenching and partitioning (Q&P) process. Wear.

